# Social Media Content on Immunology: Is an Assessment by the Scientific Community Required?

**DOI:** 10.3390/vaccines11020473

**Published:** 2023-02-17

**Authors:** Simone Morra, Francesco Di Bello, Claudia Collà Ruvolo, Gianluigi Califano

**Affiliations:** 1 Cancer Prognostics and Health Outcomes Unit, Division of Urology, University of Montréal Health Center, Montréal, QC H4A 3J1, Canada; 2 Department of Neurosciences, Reproductive Sciences and Odontostomatology, University of Naples Federico II, Via Sergio Pansini 5, 80131 Naples, Italy

In recent years, vaccines and immunotherapy have become two of the most promising and effective tools in the fight against a wide range of diseases, from the common cold to cancer. Both have the potential to save countless lives, but their impact is dependent on widespread access and adoption. Unfortunately, despite the proven safety and efficacy of these treatments, there are still many people who are skeptical or even opposed to them. In an era of information overload, where social media (SoMe) platforms are a primary source of news and information, it is critical that we take a closer look at how these platforms shape the public’s understanding of vaccines and immunotherapy.

Today, over 50 million Americans search for medical information and advice on the Internet [1]. This phenomenon is even more accentuated after the COVID-19 pandemic outbreak [2]. Specifically, we are witnessing a growing use of SoMe platforms worldwide: data from Pew Research Center report that the percentage of Americans who use SoMe rose from 5% in 2005 to 72% in 2021 [3]. Similarly, in 2021, the percentage of Europeans aged 16–74 that used SoMe was around 57%, with the highest rate reported in Denmark (85%) and the lowest in Italy (48%) [4]. Among the various platforms currently available to Internet users, the most used is YouTube. It is noteworthy that this SoMe platform represents the one with the highest rates in terms of use for both genders (which stands at around 80%), for different race/ethnicity groups (ranging from 79% for White people to 85% for Hispanic people) and, even more important, for all income groups (ranging from 75% to 90% for the population with a median annual income lower than USD 30K and higher than USD 75K, respectively) and ages (ranging from 49 to 95% for +65 to 18–29 years old, respectively) [3]. The users who mostly carry out online searches on medical topics are represented by patients suffering from chronic or oncological diseases, and their caregivers [5].

Among the chronic diseases with the highest prevalence, rheumatological diseases are to be counted. Specifically, arthritis is diagnosed in 23% of adult Americans and psoriasis affect 1–4% of the population worldwide [6,7]. Additionally, a recent report by the World Health Organization (WHO), has revealed that infectious diseases, specifically lower respiratory infections, represent the first cause of communicable death worldwide, for both males and females [8]. Moreover, among the top 10 causes of death in low-income countries, along with lower respiratory tract infections, are other infectious diseases, such as diarrheal diseases, malaria, tuberculosis and HIV/AIDS [9]. Furthermore, the relevance of infectious diseases is not negligible considering that some infectious agents have been classified as causal agents of some neoplasms [10]. Specifically, the human papillomavirus is one of the most potent viruses that causes sexually transmitted diseases in a large-scale population worldwide, and it is the major causative agent for carcinoma of the uterine cervix, squamous cell carcinoma of the penis and oropharyngeal carcinomas [11]. Additionally, chronic hepatitis B infections are responsible for more than 820,000 deaths annually due to liver cirrhosis and hepatocellular carcinoma [12]. For both of the aforementioned viruses, effective vaccines have received Food and Drug Administration approval since 2006 and 1981, respectively [13,14]. The correct implementation of a vaccination campaign would drastically reduce the spread of the diseases and the relative deaths connected to them.

Moreover, the recent phenomenon of the COVID-19 pandemic has brought to light the large impact that infectious diseases can have even in high-income countries, placing a strain on healthcare systems [15]. The important role of immunology in the fight against COVID-19 is incontestable. Given the imperative for speed, the ability to rapidly develop a vaccine was instrumental to coping with the pandemic [16].

Of no less importance for collective health, oncological pathologies must be considered. Specifically, GLOBOCAN 2020 estimates of cancer incidence and mortality produced by the International Agency for Research on Cancer estimated 19.3 million new cancer cases and almost 10.0 million cancer deaths in 2020 [17]. Although surgery and chemotherapy still represent the cornerstones of the treatment of this type of disease, immunotherapy is changing the way we think about and treat cancer. In fact, to date, there are notable applications, in various types of cancer, of drugs designed to stimulate the body’s immune response against neoplastic cells [18,19,20].

It is undeniable that SoMe platforms have often been the location of debates on medical topics, and this phenomenon has further increased during the COVID-19 pandemic. Nevertheless, the medical contents uploaded to SoMe are not subjected to any proofreading operation, meaning that the quality of the information is not guaranteed. Therefore, in the last year, as well as for other specialties [21,22,23,24,25,26,27,28,29,30,31,32,33,34], many works that analyzed the quality of immunological information on social media have been produced [35,36,37,38]. The result of the analysis of YouTube content on immunological topics is that the content is of a low-quality, and especially greatly confused in the description of the chosen topic. Moreover, it is increasingly evident that the most popular videos are not necessarily those of the highest quality. Therefore, a revision of these contents is necessary by the scientific community.

## Data Availability

Not applicable.
